# Supplementary material for: Molecular Characterization of a Clade 2.3.4.4b H5N1 High Pathogenicity Avian Influenza Virus from a 2022 Outbreak in Layer Chickens in the Philippines
Source: Pathogens. 2024 Sep 28;13(10):844. doi: 10.3390/pathogens13100844 (PMC11510588; doi:10.3390/pathogens13100844)

Supplementary Figure S1. Phylogeny for all eight segments of two virus isolates (PTY and MHN) detected in chickens from Central Luzon, Philippines.

A large database of avian influenza viruses was downloaded from the GISAID (Global Initiative on Sharing All Influenza Data) database using the following search parameters for HA: H5 clade 2.3.4.4b/c/e/g/h from avian hosts and any location; and for the rest of the segments: all sequences from Asia and from avian hosts of any subtype. These sequences were down-sampled using CD-HIT. Sequences from top BLAST hits namely, A/feline/South-Korea/SNU1/2023, A/crow/Fukuoka/TU48-37/2022, A/crow/Miyagi/TU69-55/2023 (highlighted in fuchsia), were added to the down-sampled dataset. To further identify the genotype of PTY and MHN, the following were added to the dataset: 4 sequences each from G2b, G2c, and G2d groups as identified by Takadate et al [1] and 45 reference sequences from Europe in 2020-2022 as identified by Fusaro et al [2]. Subsequently, these sequences were aligned using MAFFT. Maximum likelihood trees were created using RaxML-ng with the GTR+FO+G4m model and automatic bootstrapping. Number on the nodes indicate the bootstrap value. Only bootstrap values ≥50 are shown.

For all eight segments, PTY and MHN share the same lineage as top BLAST hits and G2c viruses, A/chicken/Nagasaki/22A6T/2022 and A/large-billed_crow/Niigata/1503B017/2023. Relative to other G2c group viruses, PTY and MHN have reassortments in the PB2, PB1, PA, NP, and NS segments. With respect to 2020-2022 clade 2.3.4.4 viruses from Europe, PTY and MHN have a distinct genotype since PTY and MHN share common ancestors with reference strains at only 3 out of 8 segments. These segments are the NP segment with reference sequence A/environment/Bangladesh/17E82/2021 (H6N1), and the NA and M segments with reference sequence A/turkey/England/057679/2021 (H5N1).

Reference

1. Takadate, Y.; Mine, J.; Tsunekuni, R.; Sakuma, S.; Kumagai, A.; Nishiura, H.; Miyazawa, K.; Uchida, Y., Genetic diversity of H5N1 and H5N2 high pathogenicity avian influenza viruses isolated from poultry in Japan during the winter of 2022–2023. *Virus Research* **2024,** 347, 199425.
2. Fusaro, A.; Zecchin, B.; Giussani, E.; Palumbo, E.; Agüero-García, M.; Bachofen, C.; Bálint, Á.; Banihashem, F.; Banyard, A. C.; Beerens, N.; Bourg, M.; Briand, F. X.; Bröjer, C.; Brown, I. H.; Brugger, B.; Byrne, A. M. P.; Cana, A.; Christodoulou, V.; Dirbakova, Z.; Fagulha, T.; Fouchier, R. A. M.; Garza-Cuartero, L.; Georgiades, G.; Gjerset, B.; Grasland, B.; Groza, O.; Harder, T.; Henriques, A. M.; Hjulsager, C. K.; Ivanova, E.; Janeliunas, Z.; Krivko, L.; Lemon, K.; Liang, Y.; Lika, A.; Malik, P.; McMenamy, M. J.; Nagy, A.; Nurmoja, I.; Onita, I.; Pohlmann, A.; Revilla-Fernández, S.; Sánchez-Sánchez, A.; Savic, V.; Slavec, B.; Smietanka, K.; Snoeck, C. J.; Steensels, M.; Svansson, V.; Swieton, E.; Tammiranta, N.; Tinak, M.; Van Borm, S.; Zohari, S.; Adlhoch, C.; Baldinelli, F.; Terregino, C.; Monne, I., High pathogenic avian influenza A(H5) viruses of clade 2.3.4.4b in Europe-Why trends of virus evolution are more difficult to predict. *Virus Evol* **2024,** 10, (1), veae027.

(A) PB2

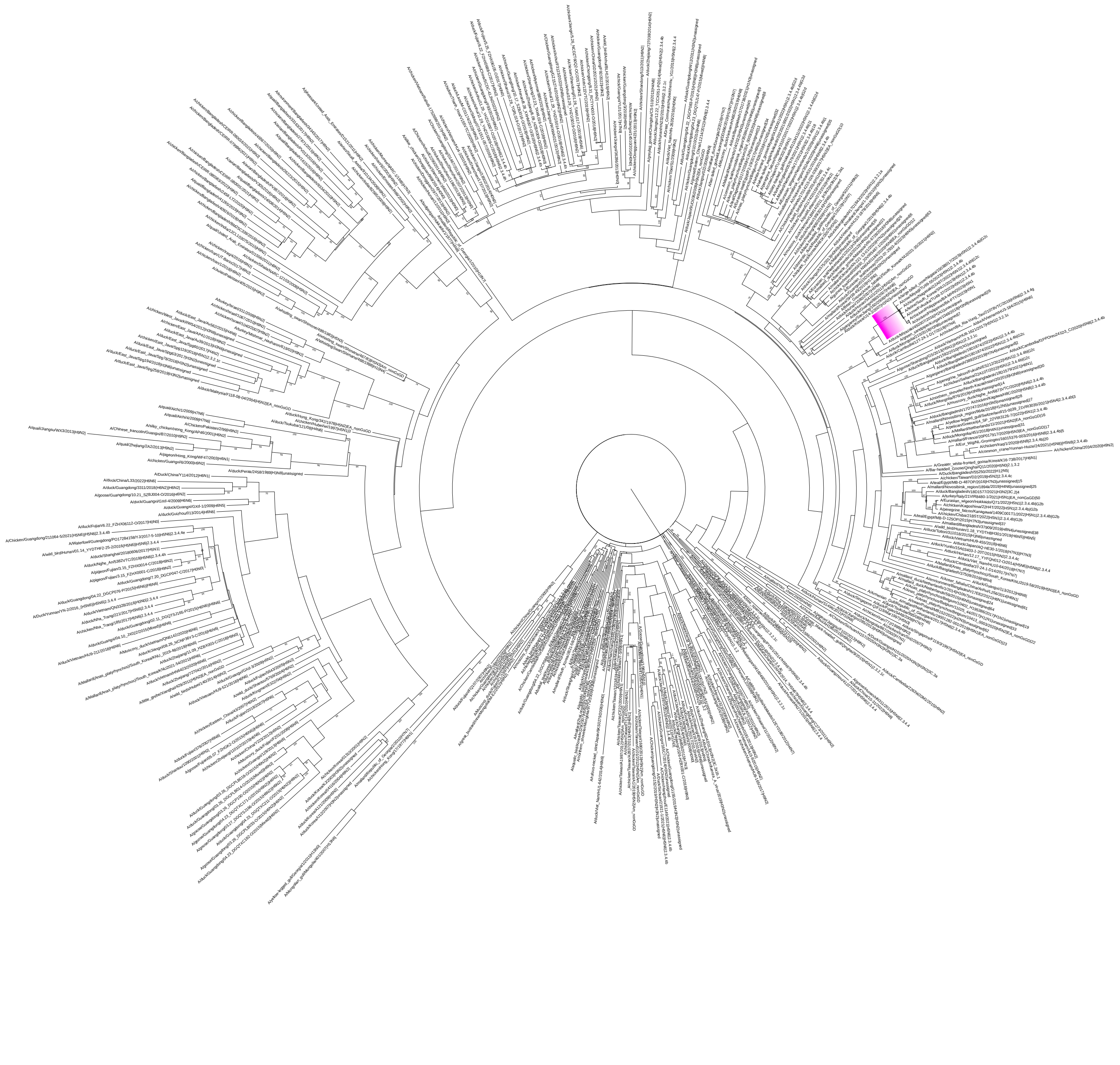

(B) PB1

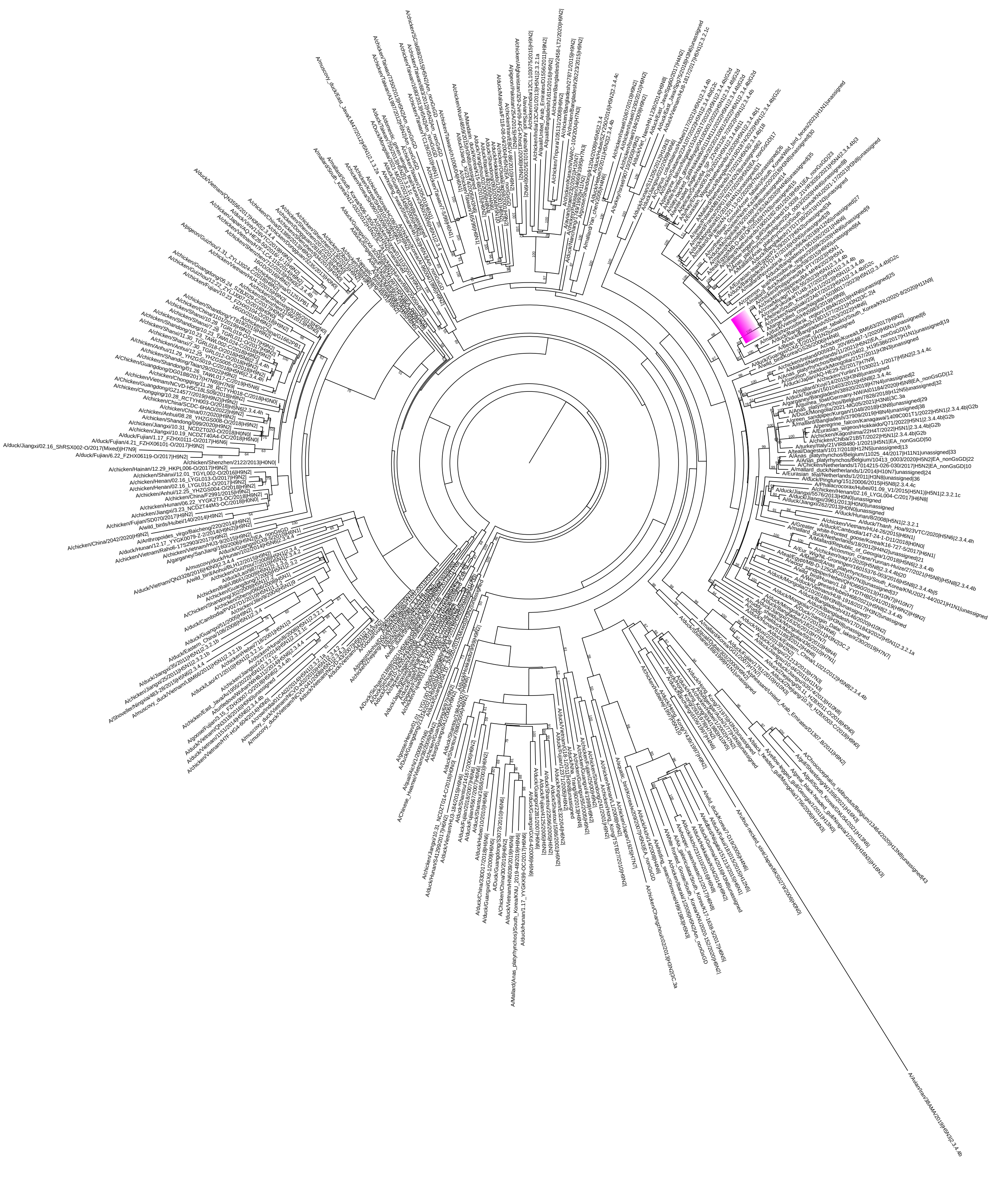

(C) PA

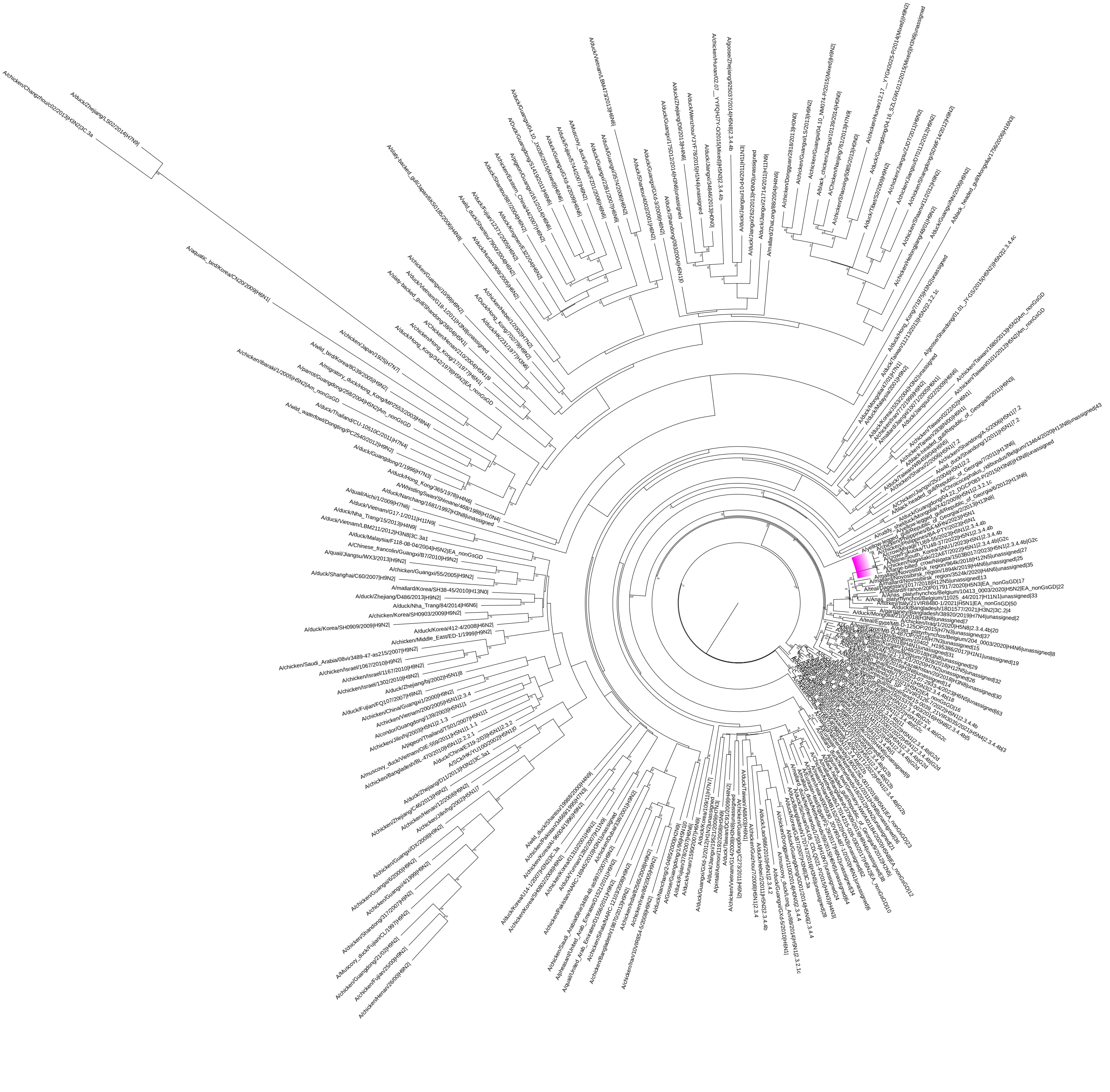

(D) HA

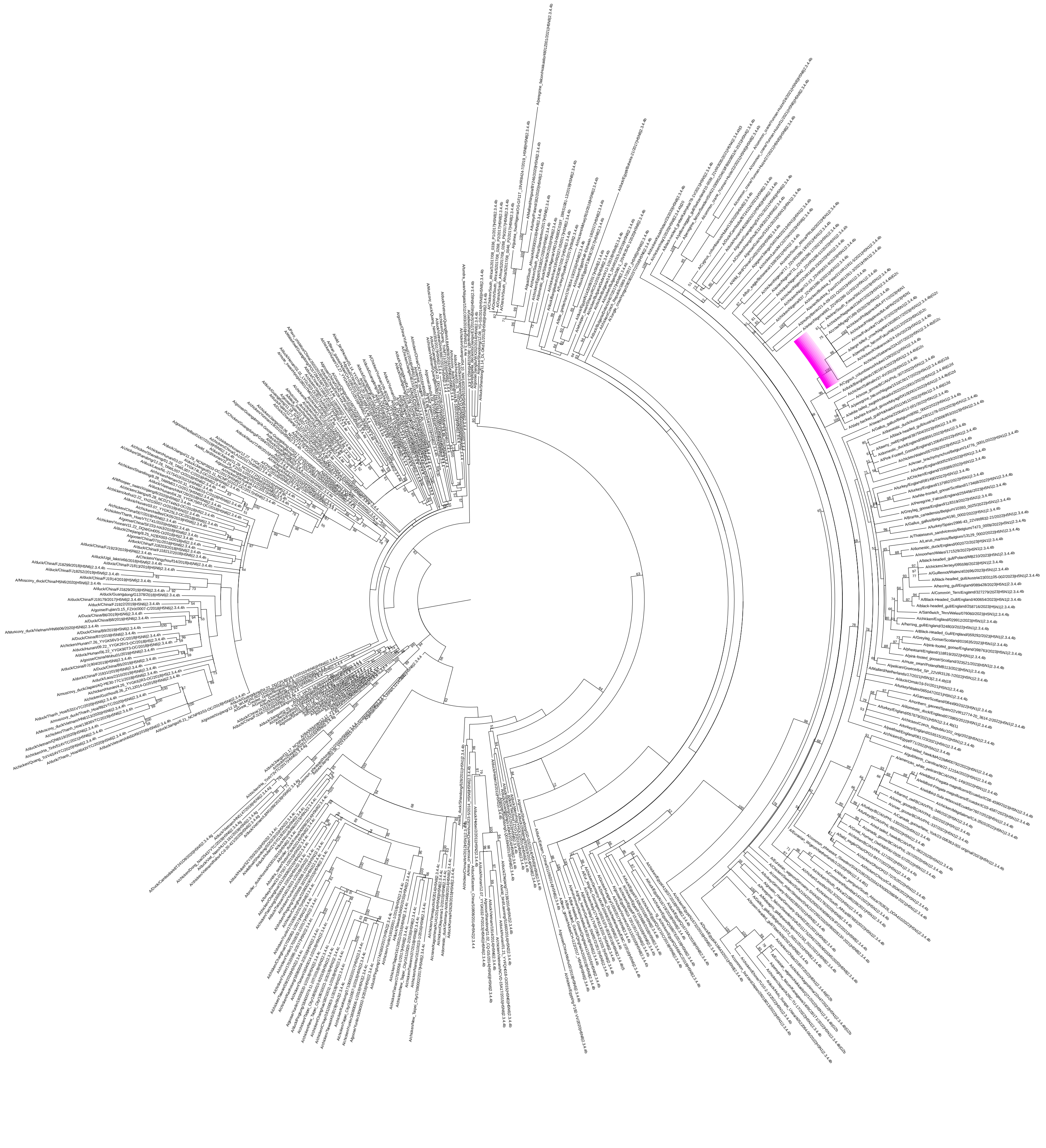

(E) NP

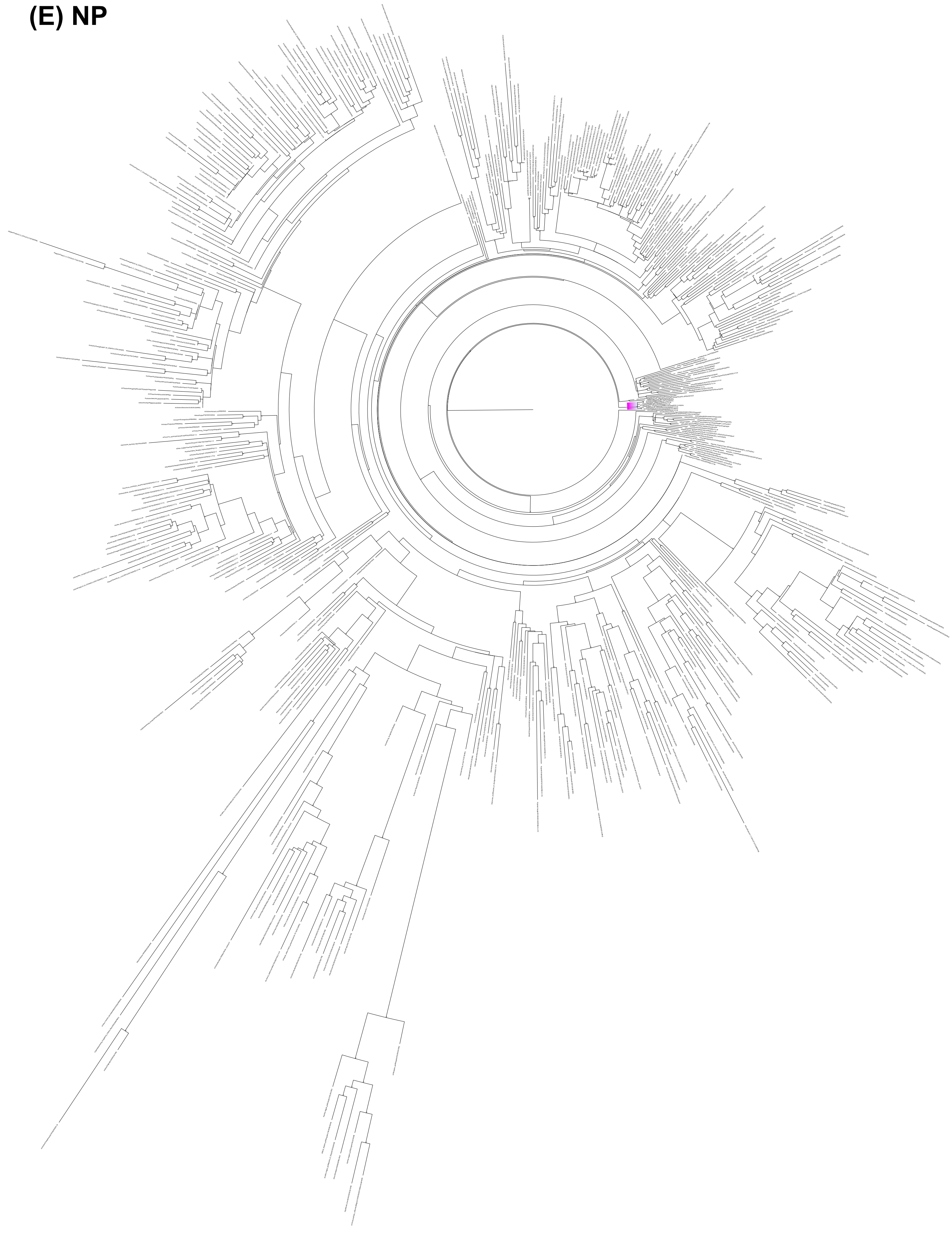

(F) NA

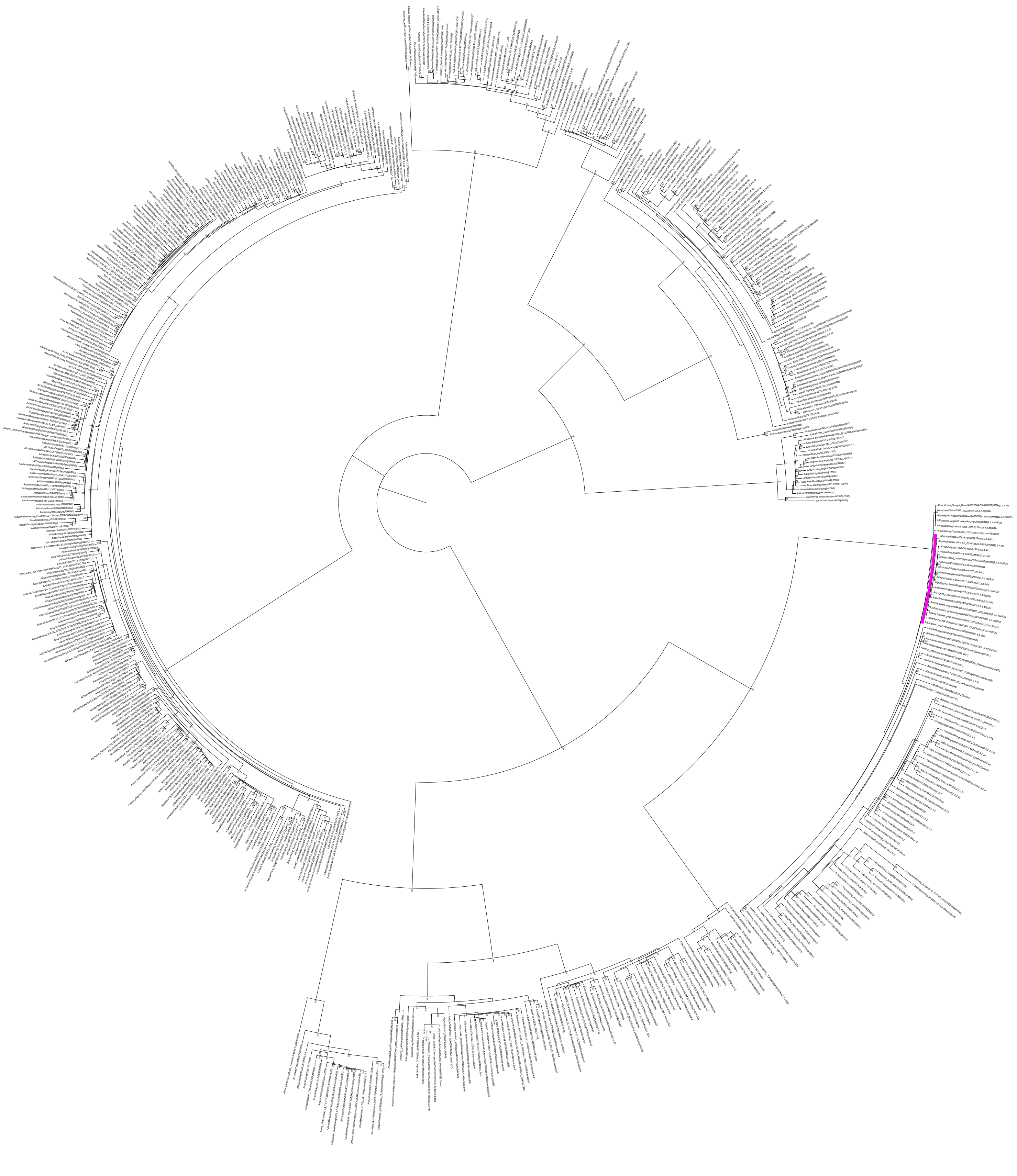

(G) M

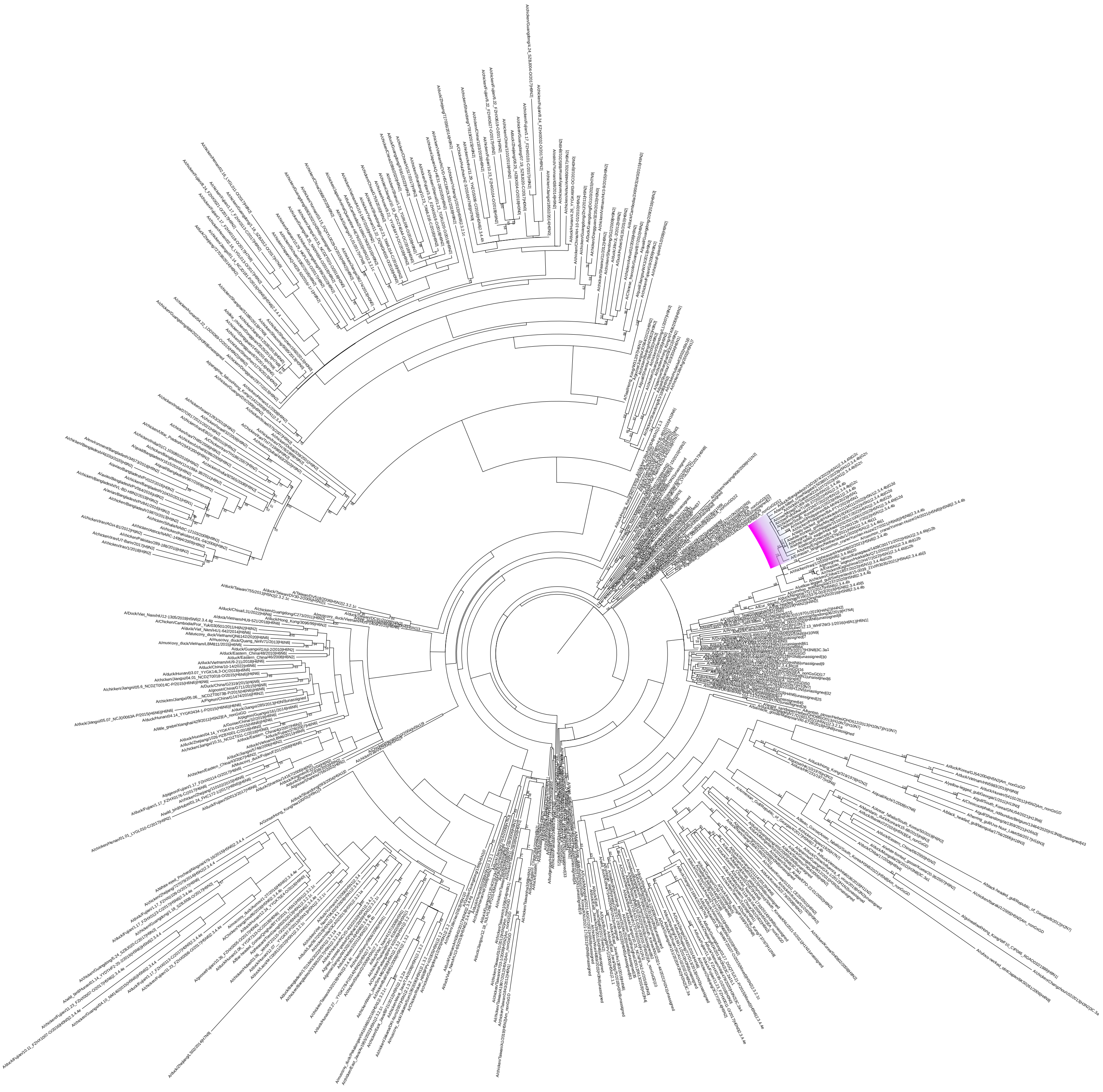

(H) NS

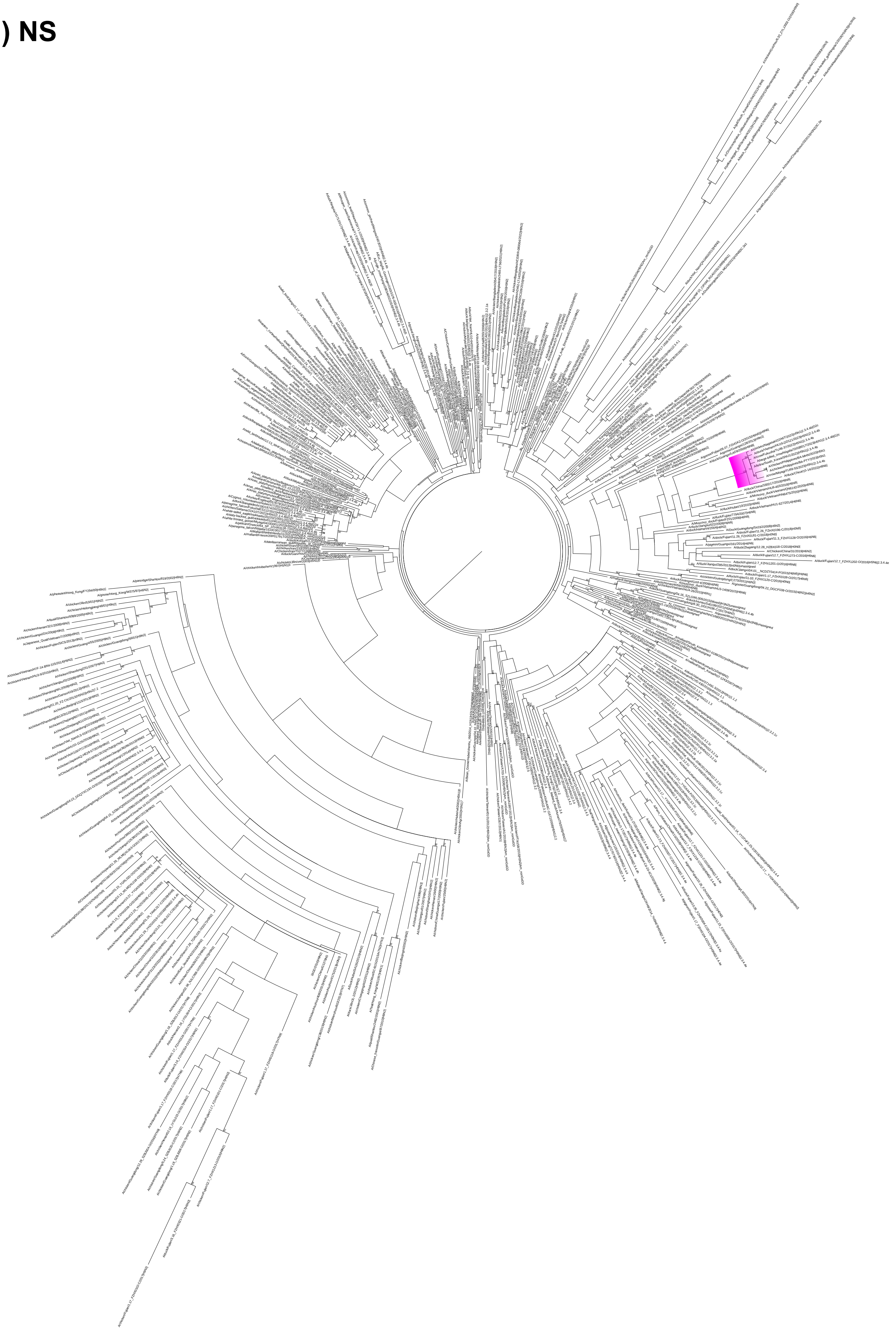

Supplement: Supplementary file 1 [file pathogens-13-00844-s001.zip › Supplementary Figure S1 Phylogeny of all segments.pdf]
