# Supplementary material for: Molecular Characterization of a Clade 2.3.4.4b H5N1 High Pathogenicity Avian Influenza Virus from a 2022 Outbreak in Layer Chickens in the Philippines
Source: Pathogens. 2024 Sep 28;13(10):844. doi: 10.3390/pathogens13100844 (PMC11510588; doi:10.3390/pathogens13100844)
Supplement: Supplementary file 1 [file pathogens-13-00844-s001.zip › Supplementary Table S1 Read counts and coverage.pdf]

Supplementary Table S1. Mean coverage and number of reads used in the reference-based assembly and consensus sequence determination of AIV from PTY and MHN samples.

	**A/chicken/Philippines/BA-PTY/2022	H5N1 (PTY)**		**A/chicken/Philippines/BA-MHN/2022	H5N1 (MHN)**	
**Segment**	*Mean Coverage*	*Number of reads used in the assembly*	*Mean Coverage*	*Number of reads used in the assembly*
**PB2**	3,547	26,300	564	4,006
**PB1**	1,376	4,956	163	854
**PA**	1,737	13,485	247	1,338
**HA**	15,883	62,375	3,892	7,614
**NP**	18,205	65,673	3,396	15,52
**NA**	10,994	36,324	3,305	18,984
**M**	49,378	176,023	5,763	26,935
**NS**	52,396	130,698	6,543	26,009
