# Supplementary material for: Molecular Characterization of a Clade 2.3.4.4b H5N1 High Pathogenicity Avian Influenza Virus from a 2022 Outbreak in Layer Chickens in the Philippines
Source: Pathogens. 2024 Sep 28;13(10):844. doi: 10.3390/pathogens13100844 (PMC11510588; doi:10.3390/pathogens13100844)
Supplement: Supplementary file 1 [file pathogens-13-00844-s001.zip › Supplementary Table S2 Molecular markers.pdf]

Supplementary Table S2. Molecular markers identified in virus isolates (PTY and MHN). These molecular markers were adapted from [1].

Viral protein	Amino acid change	Subtype examined	Phenotype	Reference
NP	M105V	H5N1	Increased clinical signs in infected chickens; Increase in replication in chickens cells; Associated with adaptation of duck viruses to chickens	[2]
NP	A184K	H5N1	Increased replication in avian cells; Increase mean death time in chickens; upregulation of pro-inflammatory genes such as IFN, nitric oxide	[3]
PB2	K389R	H7N9	Enhance growth in human and mammalian cells; enhanced polymerase activity in human cells; increased vRNA production in human cells	[4]
PB2	V598T	H7N9	Enhance growth in human and mammalian cells; enhanced polymerase activity in human cells; increased vRNA production in human cells; increased mortality, body weight loss, and replication in the lungs of mice	[4]
PB2	S715N	H5N1	Decrease mortality and mean death time in mice	[5]
HA	D94N	H5N1	Enhance virus fusion activity and increased HA binding to α-2,6 sialic acid	[6]

References

1. Suttie, A.; Deng, Y. M.; Greenhill, A. R.; Dussart, P.; Horwood, P. F.; Karlsson, E. A., Inventory of molecular markers affecting biological characteristics of avian influenza A viruses. *Virus genes* **2019,** 55, (6), 739-768.
2. Tada, T.; Suzuki, K.; Sakurai, Y.; Kubo, M.; Okada, H.; Itoh, T.; Tsukamoto, K., NP body domain and PB2 contribute to increased virulence of H5N1 highly pathogenic avian influenza viruses in chickens. *J Virol* **2011,** 85, (4), 1834-46.
3. Wasilenko, J. L.; Sarmento, L.; Pantin-Jackwood, M. J., A single substitution in amino acid 184 of the NP protein alters the replication and pathogenicity of H5N1 avian influenza viruses in chickens. *Arch Virol* **2009,** 154, (6), 969-79.
4. Hu, M.; Yuan, S.; Zhang, K.; Singh, K.; Ma, Q.; Zhou, J.; Chu, H.; Zheng, B.-J., PB2 substitutions V598T/I increase the virulence of H7N9 influenza A virus in mammals. *Virology* **2017,** 501, 92-101.
5. Sun, H.; Cui, P.; Song, Y.; Qi, Y.; Li, X.; Qi, W.; Xu, C.; Jiao, P.; Liao, M., PB2 segment promotes high-pathogenicity of H5N1 avian influenza viruses in mice. *Frontiers in Microbiology* **2015,** 6.
6. Su, Y.; Yang, H.-Y.; Zhang, B.-J.; Jia, H.-L.; Tien, P., Analysis of a point mutation in H5N1 avian influenza virus hemagglutinin in relation to virus entry into live mammalian cells. *Archives of Virology* **2008,** 153, (12), 2253-2261.
